# Application of a generative adversarial network for multi-featured fermentation data synthesis and artificial neural network (ANN) modeling of bitter gourd–grape beverage production

**DOI:** 10.1038/s41598-023-38322-3

**Published:** 2023-07-20

**Authors:** Sefater Gbashi, Tintswalo Lindi Maselesele, Patrick Berka Njobeh, Tumisi Beiri Jeremiah Molelekoa, Samson Adeoye Oyeyinka, Rhulani Makhuvele, Oluwafemi Ayodeji Adebo

**Affiliations:** 1 Department of Biotechnology and Food Technology, Faculty of Science, Doornfontein Campus, University of Johannesburg, P.O Box 17011, Johannesburg, 2028 Gauteng South Africa; 2 Food Innovation Research Group, Department of Biotechnology and Food Technology, Faculty of Science, University of Johannesburg, P.O Box 17011, Johannesburg, 2028 Gauteng South Africa; 3 National Centre for Food Manufacturing, Centre of Excellence in Agri-Food Technologies Building, South Lincolnshire Food Enterprise Zone Campus, University of Lincoln, 2 Peppermint Way, Holbeach, Spalding, PE12 7FJ Lincolnshire UK; 4 Toxicology and Ethnoveterinary Medicine, Agricultural Research Council-Onderstepoort Veterinary Research (ARC-OVR), Private Bag X05, Onderstepoort, Pretoria, 0110 Gauteng South Africa

**Keywords:** Biochemistry, Computational biology and bioinformatics

## Abstract

Artificial neural networks (ANNs) have in recent times found increasing application in predictive modelling of various food processing operations including fermentation, as they have the ability to learn nonlinear complex relationships in high dimensional datasets, which might otherwise be outside the scope of conventional regression models. Nonetheless, a major limiting factor of ANNs is that they require quite a large amount of training data for better performance. Obtaining such an amount of data from biological processes is usually difficult for many reasons. To resolve this problem, methods are proposed to inflate existing data by artificially synthesizing additional valid data samples. In this paper, we present a generative adversarial network (GAN) able to synthesize an infinite amount of realistic multi-dimensional regression data from limited experimental data (n = 20). Rigorous testing showed that the synthesized data (n = 200) significantly conserved the variances and distribution patterns of the real data. Further, the synthetic data was used to generalize a deep neural network. The model trained on the artificial data showed a lower loss (2.029 ± 0.124) and converged to a solution faster than its counterpart trained on real data (2.1614 ± 0.117).

## Introduction

Fermentation is an important food processing operation performed in the production of functional beverages and remains the only means of production of alcoholic beverages both traditionally and industrially. Alcohol is an important parameter of fermented beverages both for its physicochemical and sensory properties, microbial ecology, and psychotropic effects, as well as for regulatory and tax purposes. Alcohol content sheds light on the nature and characteristics of the fermenting microorganisms, as well as the characteristics of the food matrix. One of the oldest pieces of evidence of alcohol production dates back to about 10,000 B.C^1^. Alcohol is still the most commonly used drug in the world^2^ and has significant implications for public health and social systems.

Around the world, there are specific regulations that enforce the labeling of alcoholic beverages. In the United States, a drink is considered non-alcoholic if it has less than 0.5% vol/vol of ethanol. In Canada, however, this value is set at 1.1%^3^. In the European Union, beverages with more than 1.2% vol/vol of alcohol are labeled differently than those with less. In the Muslim world, naturally fermented foods with less than 1.0% ethanol can be considered Halal^4,5^. The consumption of alcoholic beverages is regulated and its sale is both controlled and taxed differently than non-alcoholic beverages. Because of these and the health and social effects of alcohol, it is critical to monitor alcohol production patterns in fermented beverages. This will help standardize the fermentation process and ensure appropriate/desired levels of alcohol in the beverages. Indeed, the fermentation system is quite intricate involving various process conditions such as temperature, type and amount of microorganisms, duration, moisture, etc. which influence the quality of the final product. Adequate knowledge of the overall fermentation system is important in order to improve the process and achieve the desired outcome.

Modeling is essential in the development and optimization stages of any manufacturing process in order to save the time and cost associated with traditional trial and error. Modeling helps in understanding system dynamics and predicting future process behavior under different scenarios and as such, could be useful in designing more efficient fermentation processes for improved food quality. There is potential to model the alcohol content of fermentation process using ANNs, as they have the ability to learn complex relationships in high-dimensional datasets. ANNs have become popular and are widely applied in the study of various food processing operations with great success^6–8^. ANNs are able to learn complex patterns within a multidimensional dataset without prior knowledge of the underlying process, rendering them very suitable for the modeling of food fermentation systems. Nonetheless, a major challenge with ANNs is that they require a large corpus of training data to effectively learn and converge to a better solution, which is not always available. This has been the main limiting factor in the application of ANNs in many scientific fields, particularly in food processing where the collection of such training data is often expensive, time-consuming, and laborious. Data augmentation overcomes this limitation by artificially increasing the size of data used for training based on existing data often using artificial intelligence (AI) algorithms^9^. Intelligent data synthesis offers numerous opportunities in machine learning (ML) and AI modelling of biological and bioanalytical data, which tend to be high dimensional and scarce. Augmented and/or synthetic data have been shown to increase the performance of AI and ML models in different scientific disciplines, including food science^10,11^, medicine^12,13^, remote sensing and spatial sciences^14^, engineering^9^, etc.

For example, Gao et al.^9^, described the construction of a regression-type GAN with gradient penalty to generate artificial flame images along with corresponding oxygen content labels. The synthetic images were then used to generalize a convolutional neural network (CNN). This approach successfully addresses the challenges of imbalanced and insufficient data encountered during the training of CNN regression models. The experimental results demonstrate that the proposed method achieves high accuracy in predicting the combustion oxygen content from flame images, even when dealing with imbalanced original datasets. In another study, Gao et al.^15^, introduced an advanced GAN architecture called the multiview Wasserstein GAN (MVWGAN), which was used to address the issue of imbalanced pearl classification data, with the aim of enhancing the level of automation of industrial pearl classification through deep learning methods. Indeed, the MVWGAN model could generate multiple high-quality images from different perspectives in order to balance the imbalanced datasets, particularly for the minority classes. These augmented and balanced datasets were then employed to train a multistream CNN (MS-CNN) for the purpose of pearl classification. Through experimentation, the authors demonstrated that the MVWGAN approach effectively overcame the challenges posed by imbalanced learning, leading to improved classification performance of the MS-CNN model.

In a prior study^16^, we described the production of a bitter gourd-grape beverage through the process of fermentation using a starter culture. The fruit of the bitter gourd is very nutritious and has a variety of health benefits, including anti-diabetic, antidementia, anticancer, antioxidant properties, etc.^16–18^. The aim of this work was to use ANN to model the alcohol production patterns during the fermentation of the bitter gourd-grape beverage under different conditions using ANN. As such, bitter gourd-grape beverage was produced following a controlled fermentation process using a yeast inoculum. Three fermentation conditions were monitored against the content of alcohol in the final product. The conditions monitored included concentration of fermenting organisms, incubation temperature, and time. The experiments produced a total of 21 experimental data samples, with the amount of data produced being constrained by the resources available.

The amount of data required to train an ANN depends on a number of factors, majorly (1) the complexity of the problem or system being modeled—generally the unknown fundamental function that best approximates the relationship between the input and output variables, and (2) the complexity of the learning algorithm—more specifically, the algorithm used to inductively learn the unknown underlying relational function from the specific data samples^19^. According to a general guideline, there should be at least 10 times as many data samples used to train a neural network (NN) as the number of independent variables in the system being modeled. Although there is no set number of data samples required to train an ANN, it is known that more training data (amongst other considerations such as the quality and diversity of the data) can enhance an ANN's performance and learning.

Since fermentation is a complex biochemical process that is difficult to model particularly when controlled under different conditions as is the case in this study, the 21 data samples generated from the experiments were deemed nominally insufficient to adequately train a NN. To overcome this limitation, a GAN model was developed to artificially inflate our data to be able to train the NN. This constituted the first objective of this study and was successfully achieved and described in the subsequent sections of this paper. After data synthesis, the second goal of this study was achieved by training a deep neural network (DNN) using both the original data and the synthetic data and then comparing how well the models trained on these two sets of data. Overall, the study describes the construction and application of a highly efficient deep GAN model that is able to synthesize multi-dimensional regression data from existing experimental data on the fermentative production of bitter gourde-grape wine. The significance and efficacious generalization of a DNN on the synthetic data in comparison with the experimental data is also demonstrated.

## Theoretical framework and related works

### Background

In the deep learning field, generative models have gained popularity for generating data that remarkably simulate real datasets^20^. A generative model is a computer algorithm, which learns how to produce synthetic samples from a data distribution. They are also able to distinguish between samples that belong to the distribution and those that do not. Amongst generative models, GANs^21,22^ are arguably the most powerful. GANs can produce artificial images of real-world objects that look so real that human evaluators are unable to distinguish from true images^23^. Further to this, GANs have demonstrated superiority in discriminative tasks with relatively small amounts of data available^24^, where equivalent deep neural networks (DNNs) and CNNs would require substantially more training data to achieve a similar level of performance (accuracy). Considering one of the biggest challenges in deep learning is obtaining the relatively large amount of labelled training data to generalize such models, the possibility of training these models with much less data is immensely important^20^.

### ANNs modelling

**Figure 1:**
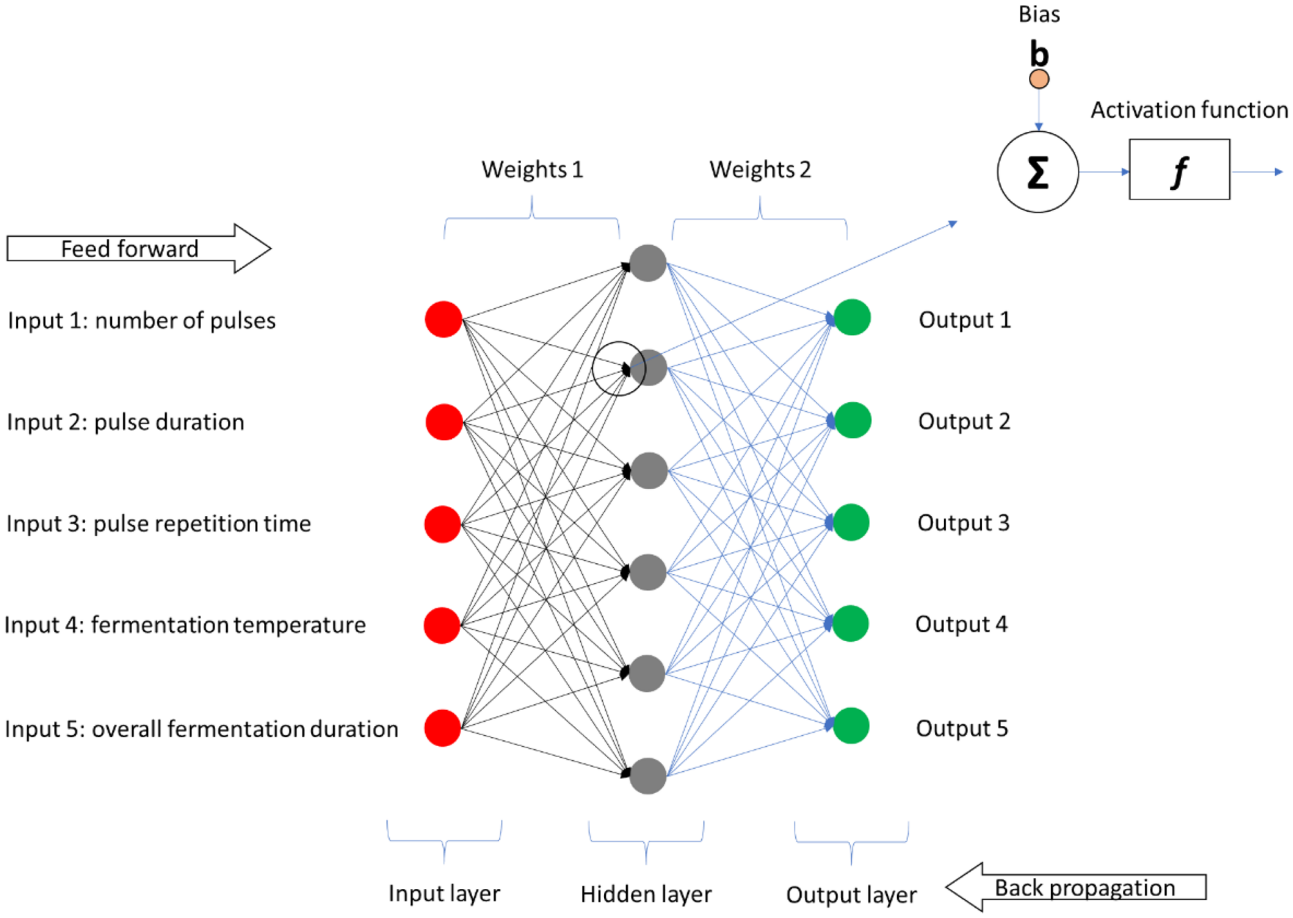
Schematic representation of a fully connected MLP neural network.

Artificial neural networks (ANNs) use the processing principle of the brain as a basis to model complex patterns and prediction problems^25^. These algorithms are essentially useful in nonlinear empirical modelling problems, inspired by biological NNs that can implicitly “learn” complex dynamic behaviours of physical systems. ANNs are able to learn all possible interactions between process variables useful in making functional approximations, recognizing patterns, and forecasting^26,27^. More recently, ANNs are enjoying a resurgence and have been applied in the study of various food processing operations including fermentation^6^, brewing^28^, cooking^29^, drying^30^, and crystallization^31^. Generally, the network architecture (i.e., specific arrangement of the layers and nodes in the network) has an input layer, hidden layer (can be more than one) and an output layer. This kind of architecture is often referred to as the multi-layer perceptron (MLP) because of the multiple layers present (Fig. 1). The architecture on an ANN largely determines the functional behaviour of the network and is specific for each application^26^. The most common configuration of MLPs is the fully connected neural networks (FCNNs) which implies that all neurons/nodes in a layer is connected to every neuron in the next layer. The major advantage of FCNNs is that they are “structure agnostic” (i.e., there are no specific assumptions to be made regarding the input features) as such, they are broadly applicable.

Inputs (or the raw data) are fed into the input layer sometimes called the visible layer as it brings the initial data into the system for further processing by subsequent layers of network (note, the input layer is the very beginning of the workflow for an ANN). Each node in the layer provides an output value via an activation function which is subsequently used as inputs to the next hidden layer. Essentially, a node (also called a perceptron or neuron) is a computational unit that has one or more weighted input connections. The nodes are organized into layers to comprise a network. The connections between nodes are calculated values referred to as weights. The weights represent the “strength or importance” of a connection between neurons and are combined in some way by a transfer function. The hidden layer(s) are situated between the input and output layers, where neurons take in a set of weighted inputs and produce an output via an activation function. This layer can be likened to a “distillation layer” that distils some of the relevant and important patterns from the inputs (leaving out the redundant information) and passes such information onto the next layer. The activation function captures nonlinear relationship between the inputs and converts it into a more useful output^25^. The output layer produces the final results (i.e., it is the last layer of neurons that produces given outputs for the program).

A good ANN model with high accuracy makes predictions that are very close to the observed values. Obtaining a good model is to find “optimal values of weights” that minimizes the prediction error. This is achieved via the “back propagation algorithm”, making ANN an algorithm with the ability to learn because it learns from the errors and the model accuracy is improved. The most common optimization algorithm is the “gradient descent” also called steepest descent, which is widely employed for minimizing cost or loss functions. Gradient descent is a first-order iterative optimization algorithm utilized for identifying a local minimum of a differentiable function and operates by taking successive steps in the direction opposite to the gradient (or an approximation of the gradient) of the function at the current point, as this represents the path of steepest descent. The gradient is the slope of the cost/loss function. The sum of squared error is usually used as a loss function. The gradients are calculated in a backward direction, from the output layer to the input layer^25,32^. Some important advantages of ANNs are that they can learn and model nonlinear and complex relationships, they can generalize (i.e., they can infer unseen relationships in data), they do not impose any restrictions on the input variables such as how they should be distributed. This makes ANNs perform very well in modelling heteroskedasticity, i.e., data with high volatility and non-constant variance^25^.

Generally, ANNs are flexible algorithms able to decipher complex nonlinear relationships between the input and output features through a learning process. This power and flexibility come at the cost of requiring a lot more training data and their performance strongly depends on the specific data used for their training. While the amount of training and testing data is not specified, it is known that with an adequately designed model, the accuracy of prediction improves with the amount of relevant training data^33^. Since ANNs are nonlinear, they require much more data to learn the patterns in the problem under study. However, availability of various biological and biochemical experimental data is limited due to analytical cost, ethical considerations, and other factors such as lengthy and tedious analytical methods, various safety hazards associated with acquiring such data, and in some cases not feasible.

### Data augmentation/synthesis

A promising solution to the challenge of limited data in ML and AI techniques is data augmentation/synthesis, which encompasses methods and techniques employed to increase the amount and/or quality of training data, or sometimes artificially generate new training datasets from existing data. Data augmentation has been shown to significantly increase the accuracy of ANNs even with small training datasets^34^. For that reason amongst others, data augmentation has been widely applied in other ML application fields such as video processing^35,36^, image processing^9,37^, biometrics^38^, medical diagnostics (e.g., chest X-ray)^39^, DNA analysis^40^, defect detection (in polymer composites)^41^, and text analysis^42^ to name a few, but sparingly applied in food science.

### Generative adversarial networks (GANs)

**Figure 2:**
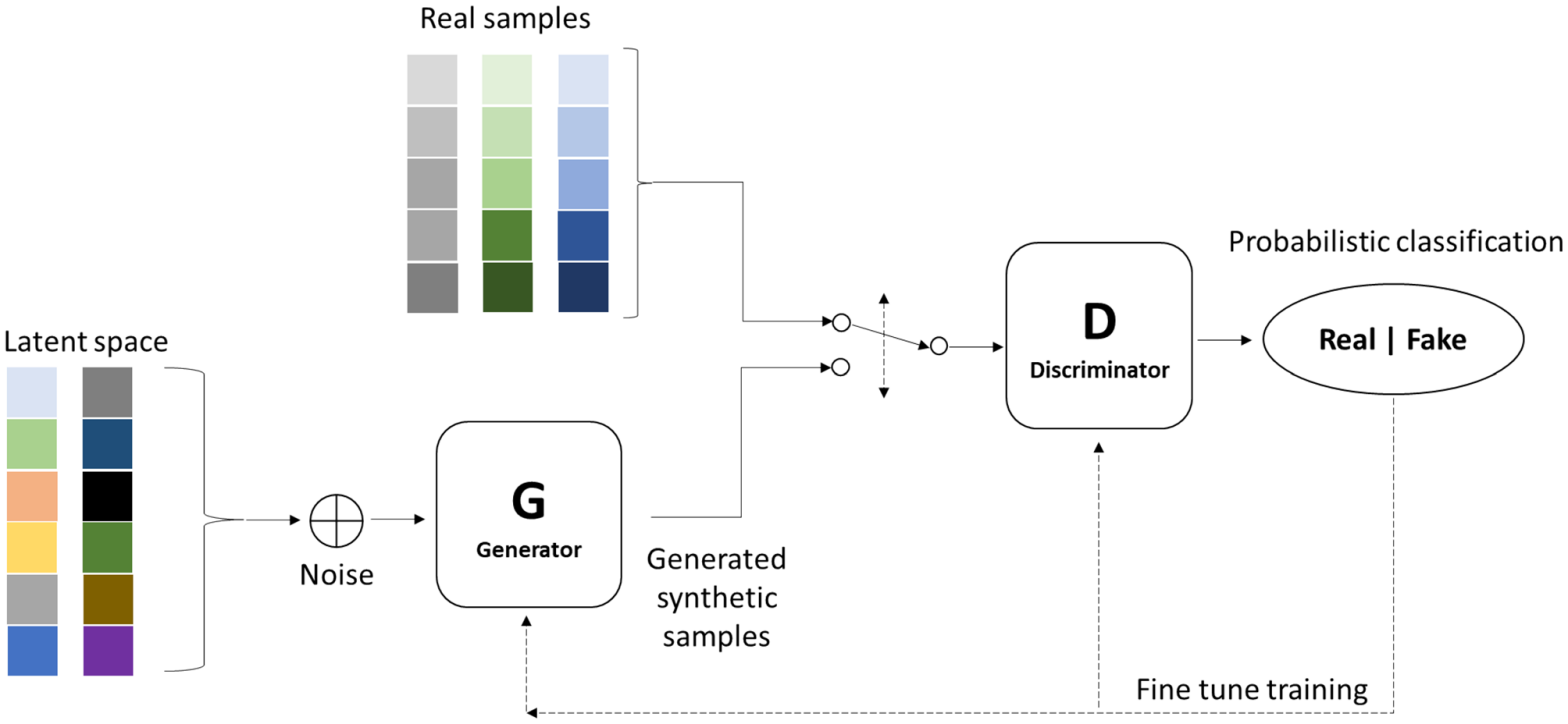
General structure of a generative adversarial network (Adapted from Gharakhanian, 2017).

Generative adversarial networks (GANs) were first introduced in 2014 by Goodfellow et al.^22^ and have increasingly gained popularity amongst researchers in different disciplines. They are fundamentally based on the concept of two analogous algorithms (a generator and a discriminator) that are adversaries perpetually attempting to outperform each other and in the process improving themselves continually^43^. In a GAN configuration (Fig. 2), the generator synthesizes artificial data, while the discriminator is tasked with the responsibility of identifying the synthetic data as either real (original) or fake (synthetic/artificial). The discriminator is usually trained on real data samples in an unsupervised approach.

As a next step, random data (noise) drawn from an n-dimensional space is then fed as input to the generator network, where it attempts to generate new data samples based on how the data points are distributed in the input samples, thereby ‘deceiving’ the discriminator with the synthesized data as real data samples. These data synthesized by the generator are then fed as input to the discriminator that classifies them as either synthetic or real. The classification error is estimated in the discriminator and the model parameters (weights) are adjusted accordingly. The same error is also backpropagated to the generator, where it attempts to maximize this error by generating more and more realistic samples. Both models attempt to fine-tune their parameters and become better in what they do^44^. If the discriminator makes the right prediction (by correctly identifying the synthetic data and the real data), the generator updates its parameters to generate better synthetic samples in order to deceive the discriminator. Also, if the discriminator makes the wrong prediction, it is penalized and as such, attempts to learn from its mistake to avoid a similar occurrence in future predictions. This process continues until an equilibrium is established and the discriminator’s training is optimized^44^. At the end of training, the generator is able to synthesize fake data that are almost identical to the original data^45^.

Since their introduction, many different GAN architectures have been proposed and intensively researched on to improve their efficiency and adaptability in different use cases such as in generating image from text^46^, completing an incomplete image^47,48^, image enhancement^49^, etc. Examples of some GAN architectures that have been reported in the literature include Fully Connected or Vanilla GAN^22^, Deep Convolutional GAN (DCGAN)^50^, Wasserstein GAN (WGAN)^51^, MVWGAN^15^, Least Square GAN (LS-GAN)^52^, Bidirectional GAN (BiGAN)^53^, etc. In consonance with existing trends, GANs have mainly been applied for image processing/classification problems, but seldom in the field of food science and technology.

Wang et al.^54^ constructed a GAN with a constraint of mean square error (MSE-GAN) to increase the original experimental data obtained during solid-state fermentation of Chinese liquor. The augmented data was subsequently utilized for training of a FCNN, which demonstrated greater accuracy in the prediction of alcohol in the liquor as compared to other prediction methods^54^. Using multiple imputation ensemble (MIE) and a Wasserstein GAN with gradient penalty, Hazra and Byun^55^ generated synthetic fermentation data of a traditional South Korean beverage, *shindari*, consumed by the people of Jeju island. In a different study, Yang et al.^56^ demonstrated the implementation of a GAN model for distinguishing fennel and cumin obtained from three different regions of China, viz Dezhou, Yumen, and Turpan. The results of the study showed that when training data was limited, the GAN model was more accurate compared to other multivariate and ML/AI classification models, with its classification accuracy even reaching 100%. Several studies have provided evidence that GANs have significant potential in supervised training, greatly improving the training of NNs, nonetheless, most of their applications have been within the jurisdiction of classification problems, with limited studies on regression problems. In this study, we propose generalizing a deep GAN model to generate regression-type data on the fermentative production of a bitter gourde-grape wine, subsequent to deep learning modelling using the synthetic data.

### Contribution

In this paper, we describe the construction and application of a multidimensional regression-type deep GAN model for the investigation of alcohol production patterns in fermented foods. The model architecture presented here has never before been described for such applications. The vast majority of GAN models and architectures are developed and utilised for classification tasks, including image augmentation and generation. Typically, regression-type GAN models are one-dimensional. However, in this work, we present a multidimensional regression-type GAN architecture capable of producing a limitless amount of multivariate synthetic data from a very small amount (n = 20) of real data. Indeed, our work is one of few that provides a framework that could guide future researches in the application of GANs within the regime of regression problems. Moreover, our GAN model can be easily modified and adapted to a range of regression-type problems, thus offering very promising prospects.

## Methodology

### Materials

Bitter gourd (*Momordica charantia*)^16^ fruit was obtained from Limpopo Province of South Africa. All other chemicals and reagents utilized in this study were of analytical grade and purchased from accredited suppliers within South Africa.

### Methods

#### Production of bitter gourd-grape wine

Bitter gourd-grape wine was produced by blending bitter gourd fruit with grapefruits in the ratio of 36:64 (w/w) in a sterile laboratory blender (Milex, Sandton, South Africa). The compositional ratio of the fruits was determined based on preliminary experimental trials. The mixture was then inoculated with yeast (*Saccharomyces cerevisiae*)^16^ and allowed to ferment. After fermentation, the substrate was filtered using a polypropylene filter cloth and immediately analysed.

#### Process monitoring and collection of experimental data

Batch fermentation experiments were performed (Supplementary Table 1) and various fermentation conditions that affect microbial metabolism, growth dynamics, and the quality of the liquor were monitored. The parameters monitored included fermentation time, temperature, and starter culture dosage. In total, 20 different experimental conditions. Following fermentation, the alcohol content of the wine was measured using a digital refractometer (Hanna Instruments (Pty) Ltd., Johannesburg, South Africa) by placing 1 ml of the wine on a sample well and recording the reading.

#### Data pre-processing

For specific use cases, it was necessary to normalize the ranges of data features in order to improve model learning/performance and to prevent violations of assumptions of normal distributions. Particularly for network training, it was critical to ensure that data features are almost on the same scale so that each feature has equal importance during the learning process, which ultimately enhances training stability and accuracy. The Scikit-learn MinMax scaler algorithm^57^ was used to standardize the data by scaling and translating each feature individually to the range of − 1, and 1.

### Generative adversarial neural network construction and validation

An adaption of the regression GAN model described by Brownlee^58^ was implemented in this study to synthesize multi-dimensional continuous fermentation data for bitter gourde-grape wine production. The data utilized for the GAN implementation was a small dataset (n = 20) obtained from experiments. The data (i.e., experimental data) was feature-scaled using the Scikit-learn MinMax scaler algorithm and its labels set to one (1) indicating that these are real samples (Supplementary Table 1^1^. Feature scaling was particularly critical because the adopted error backpropagation (i.e., gradient descent) algorithm is sensitive to the scale of data features.

**Figure 3:**
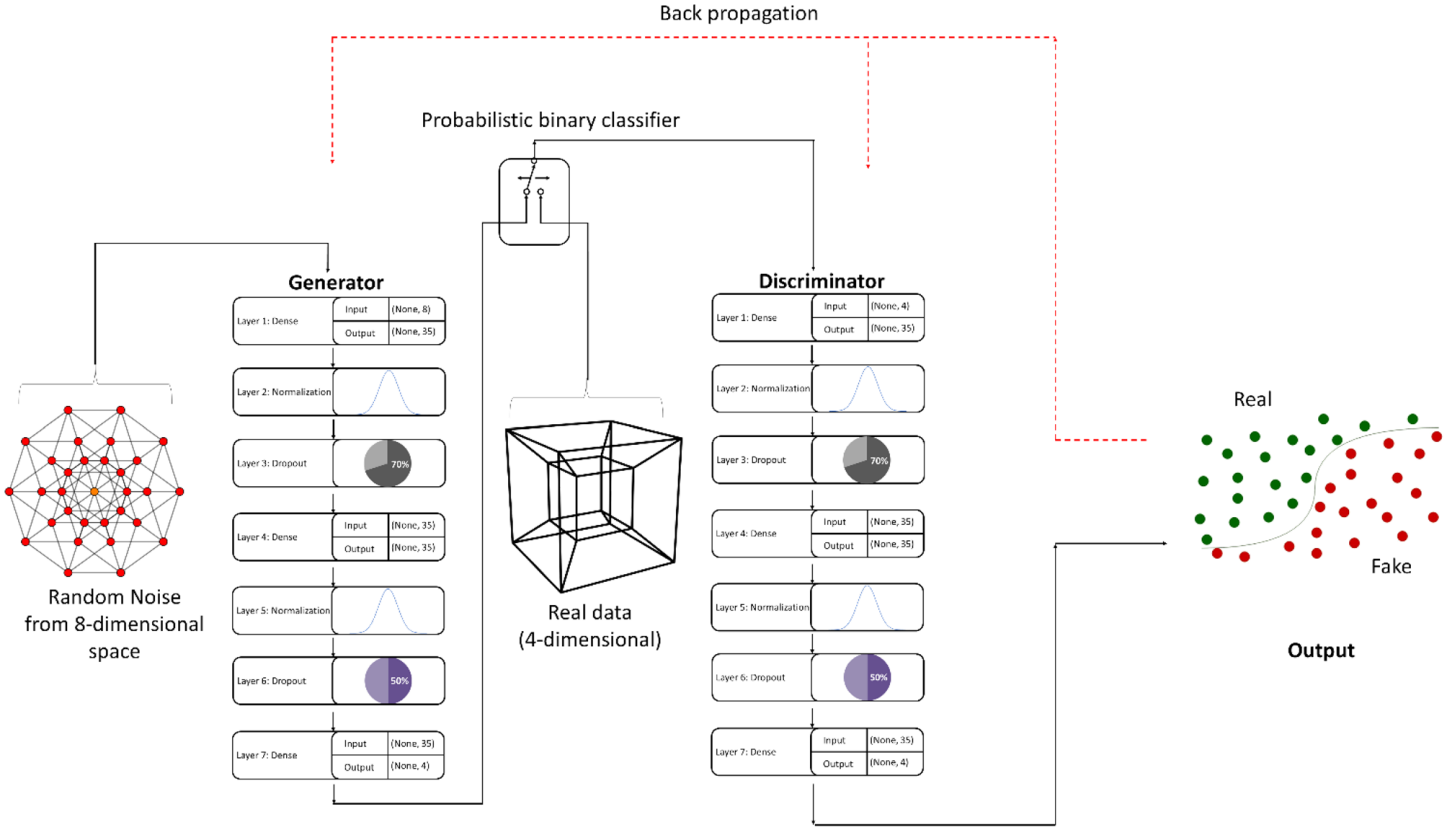
Schematic representation of the GAN architecture.

Fundamentally, the GAN model (Fig. 3) consisted of a generator and a discriminator. Details of the model configuration are presented in Table 1. The generator was supplied with another set of data labelled zero (0) indicating it is random noise which was sampled from a multi-dimensional vector space, specifically, an eight-dimensional latent space. As the generator learns the distribution patterns in the real dataset, it begins to assign meaning to points in this previously meaningless latent space. After training, the generator outputs new synthetic data. The discriminator was assigned the duty to judge the probability that the generated synthetic data was real (i.e., experimental) or fake (i.e., synthetic) and output a binary classification in this regard. The Adam version of stochastic gradient descent was used to update the network weights iteratively during model training. The model’s objective was to minimize the binary cross entropy loss function, and its performance was evaluated using mean square error (MSE) metric. The training iteration period was set to 10,000 epochs, the batch size was 10, and the model was evaluated after every 20 training iterations. These parameters were chosen based on several trial experiments.

**Table 1:** GAN model architecture and configuration.

S/No	Model	Parameters
1	**Discriminator model**	**Sequential**
	Layer 1	Dense [Input dimensions = 4, No. of nodes = 22, Activation = relu, Kernel initializer = he_uniform, Kernel regularizer = l1(0.1), Bias regularizer = l1(0.1), Activity regularizer = l2(0.1)]
	Layer 2	Batch Normalization
	Layer 3	Dropout = 0.7
	Layer 4	Dense [No. of nodes = 35, Activation function = sigmoid, Kernel regularizer = l1(0.1)]
	Layer 5	Batch Normalization
	Layer 6	Dropout = 0.5
	Layer 7	Dense [Output dimensions = 4, Activation function = sigmoid]
	Model compilation	Loss function = binary cross entropy
		Optimizer = Adam [learning rate = 1 × 10^–5^, beta_1 = 0.8, beta_2 = 0.8, epsilon = 1 × 10^–2^, clipnorm = 1 × 10^–4^]
		Metrics = mean squared error
2	**Generator model**	**Sequential**
	Layer 1	Dense [Input dimensions = 8, No. of nodes = 22, Activation = relu, Kernel initializer = he_uniform, Kernel regularizer = l1(0.1), Bias regularizer = l1(0.1), Activity regularizer = l2(0.1)]
	Layer 2	Batch Normalization
	Layer 3	Dropout = 0.7
	Layer 4	Dense [No. of nodes = 35, Activation function = sigmoid, Kernel regularizer = l1(0.1)]
	Layer 5	Batch Normalization
	Layer 6	Dropout = 0.5
	Layer 7	Dense [Output dimensions = 4, Activation function = tanh]
	Model compilation	Loss function = binary cross entropy
		Optimizer = Adam [learning rate = 1 × 10^–5^, beta_1 = 0.8, beta_2 = 0.8, epsilon = 1 × 10^–2^, clipnorm = 1 × 10^–4^]
		Metrics = mean squared error
3	**GAN model**	Sequential [Discriminator trainable = false]
	Layer 1	Generator
	Layer 2	Discriminator
		Loss function = binary cross entropy
		Optimizer = Adam [learning rate = 1 × 10^–5^, beta_1 = 0.8, beta_2 = 0.8, epsilon = 1 × 10^–2^, clipnorm = 1 × 10^–4^]
		Metrics = mean squared error

### GAN model validation

The fidelity of our GAN was determined by rigorously testing whether the model is able to learn the distribution patterns of the fermentation data and synthesize data with similar characteristics. Symbolic representations of the data patterns are made available on generated 3D surface plots of the datasets following a second-order optimization model fit using the Scipy optimization curve fit algorithm in Python programming language. The Welch’s and Brown-Forsythe tests were performed to evaluate for significant differences in population means between the two datasets. The Kruskal–Wallis H test was employed to check whether the features from the two datasets originated from the same distribution. These tests i.e., Welch’s, Brown-Forsythe, and Kruskal–Wallis tests were performed using IBM SPSS statistical software version 22 (SPSS Inc., Chicago, Illinois, USA). The datasets were also scrutinized for inherent discriminatory patterns using advanced multivariate models, principal component analysis (PCA) and orthogonal projections to latent structures discriminant analysis (OPLS-DA), performed using the SIMCA-P^+^ 16.0 chemometrics software (Umetrics, MKS Instruments Inc., Sweden)^59^.

### ANN modelling of the fermentative production of bitter gourd-grape wine

A fully connected feed-forward DNN (Table 2) constructed in Python programming language was used for modelling of the fermentative production of bitter gourde-grape wine. The algorithm consisted of seven layers (1 input layer, 5 hidden layers, and an output layer). The number of nodes in the input and hidden layers were systematically optimized using the hyperband hyperparameter optimization algorithm. The output layer contained a single node because the data had only one output feature (dependent variables). The training epochs and batch size were optimized using experimental trials. The activation functions and other model hyperparameters were also systematically optimized using the hyperband hyperparameter optimization algorithm.

**Table 2:** Deep learning model architecture and configuration.

Model	Parameters
Layer 1	Dense [No. of nodes = 11, Activation = softmax, Kernel initializer = he_uniform, Kernel regularizer = l1(0.1), Bias regularizer = l1(0.1), Activity regularizer = l2(0.1)]
Layer 2	Batch Normalization
Layer 3	Dropout = 0.6
Layer 4	Dense [No. of nodes = 6, Activation function = softmax, Kernel initializer = he_uniform, Kernel regularizer = l1(0.1), Bias regularizer = l1(0.1), Activity regularizer = l2(0.1)]
Layer 5	Batch Normalization
Layer 6	Dropout = 0.3
Layer 7	Dense [Output dimensions = 1, Activation function = relu]
Model compilation	Loss function = mean squared error
	Optimizer = Adam [learning rate = 1 × 10^–2^, beta_1 = 0.9, beta_2 = 0.999, epsilon = 1 × 10^–7^, amsgrad = False]
	Metrics = mean squared error

Limited training data might result in an underfit model amongst other problems. Essentially, the model will have a high error rate on both the training set and unobserved data because it is unable to effectively learn the relationship between the input and output variables. As such, we utilized the GAN algorithm to synthesize 200 artificial data samples from the same distribution as the original data. Both the synthetic data and original data were utilized for training the DNN. To avoid the “overtraining or overfitting” phenomena, the training data was split into three, i.e., training set (sub-sample of data used to fit the model), validation data (sub-sample of data used to provide an unbiased evaluation of model fit on the training dataset while tuning model hyperparameters), and test data (sub-sample of data held out to give an unbiased assessment of how well the final model fits the training dataset). The training set constituted 58% of the data, the validation set constituted 38% of the data, while the test set constituted 4% of the data. The training data was sampled randomly to train the model and the performance recorded on the validation set. The model performance was evaluated according to the mean square error (MSE) between the predicted and experimental values.

## Results and discussion

### Bitter gourd-grape beverage

The fermentation process is a complex biochemical process that involves multiple variables with different properties which needs to be adequately optimised in order to obtain beverages of desired quality. A functional low alcoholic beverage was produced in this study by fermenting a composite of bitter gourd fruit and grapefruits under different statistically pre-designed experimental conditions. The health benefits of this beverage including antioxidant and antidiabetic properties and the flavonoid and phenolic contents have been demonstrated in our previous study^16^. Herein, we monitored the alcohol content of the fermented beverage under 21 different experimental conditions which ranged from 0.9 to 11.8 (°P). The highest alcohol content was observed when the fruit biomass was fermented at 45.11 °C for 72 h using a starter culture dosage of 3 3.00 v/v, while the lowest alcohol content was recorded when the biomass was fermented for 72 h at 35.5 °C without using any starter culture.

The amount of alcohol in beverages is very important for the mouth-feel and flavour^60^. The alcohol content of a fermented food can also provide insight into the nature and characteristics of the fermenting microorganisms. It can also be used as a taxation factor as well as to measure the quality of alcoholic beverages. Given the legal, regulatory, and health significance associated with the alcohol content of fermented beverages available to the public, it was critical to investigate the alcohol production patterns of bitter gourd-grape beverage in this study. Modeling of alcohol production patterns during fermentation could aid in diversifying beverages and meeting specific consumer or regulatory demands to produce a variety of drinks, such as low or high alcohol beverages. Nonetheless, to construct effective models able to address the complexity of real-life problems, a vast amount of training samples are necessary, as was the case in this work. To compensate for the lack of data, we constructed a GAN model to learn the dimensional and distributional patterns in the 21 samples of experimental data we collected, and then synthesise high-quality data from the same space. The subsequent section of this article describes the training of the GAN model and its efficacy in data synthesis.

### Generation of synthetic data

A GAN model was constructed to adequately inflate experimental fermentation data on bitter gourde-grape wine production in order to model the fermentation system. The proposed GAN architecture and configuration are described in Table 1. Both the generator and discriminator of our GAN model are fully connected DNNs with optimized hyperparameters. After 10,000 training iterations, the mutual antagonistic learning of the GAN’s generator and discriminator algorithms were sufficiently equilibrated. The model could thus adequately explore the domain of the real data and synthesize artificial data with markedly similar distribution patterns as the original experimental data. Using the GAN model, we generated high quality data in a sufficient quantity (i.e., tenfold the size of the training set) that still preserved the structure, variance and other distributional features of the original data without any specific assumptions about the nature of the probability density functions of the data features (Supplementary Table 2). Moreover, our proposed GAN has the capacity to generate an infinite number of synthetic data, and can easily be adopted to similar regimes of regression problems in AI and ML, which provides great prospects to the field considering that most of the GAN architectures are for classification-type problems.

### GAN model validation and estimating the quality and fidelity of the synthetic data

**Figure 4:**
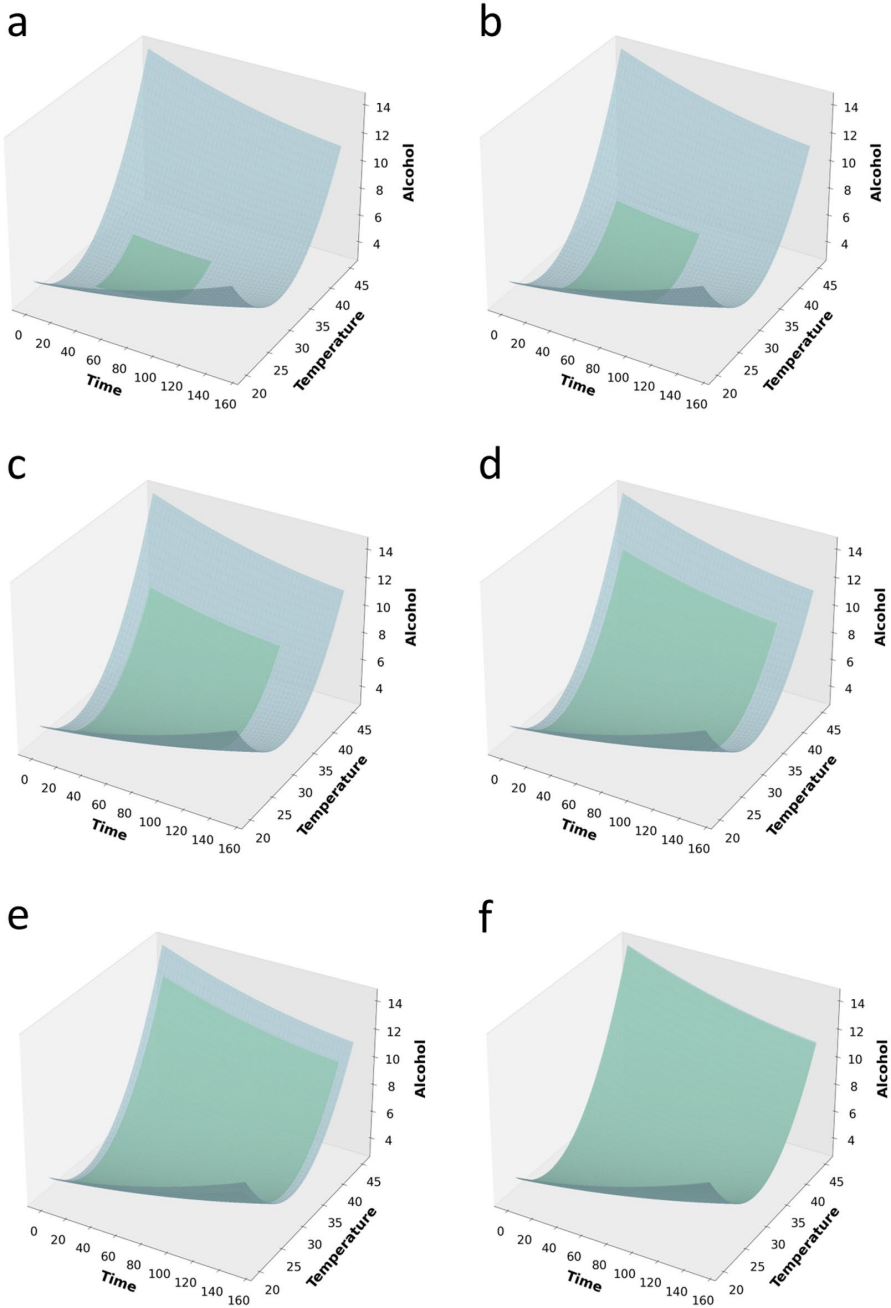
Overlayed surface plots showing the spatial distributional patterns of the datasets at selected training epochs: (**a**) 0 epoch, (**b**) 40 epochs, (**c**) 100 epochs, (**d**) 140 epochs, (**e**) 200 epochs, and (**f**) 10,000 epochs. X-axis = temperature (°C), y-axis = time (h), z-axis = alcohol content (°P), at constant culture dosage of 3.18 (v/v).

A critical part of data synthesis is the evaluation of the similarity between the generated data and the original data. A visual comparison of the distribution profiles of the real data and the synthetic data can be seen on the 3D surface plots (Fig. 4). These plots are an overlay of two surface plots in one. The blue surface shows the 3D spatial distribution profiles of the real data while the green surface shows the 3D spatial distribution profiles of the synthetic data at mean starter culture dosage. These plots clearly reveal a steady and progressive learning process for the GAN. In Fig. 4f (i.e., surface plot of real and synthetic data after 10,000 training epochs), it can be seen that it is almost impossible to visually distinguish between spatial distribution of the real data and the synthetic data generated by the GAN. This provides strong evidence that the GAN significantly approximated the distribution patterns in the real data. Of course, a good synthetic data should have similar statistical and distribution characteristics as the original data. In the literature, GANs have produced super realistic samples that were practically indistinguishable from the original samples^23,61^. For example, in a study by Zhou et al.^62^, the authors designed a GAN that generated/manipulated image samples, which fooled an AI-based computer-aided diagnosis (CAD) algorithm for breast cancer. The synthetic images fooled the AI-CAD algorithm to wrongly diagnose over 69% of cases in which it previously was able to correctly analyse. This attests to the incredible power of GANs in learning complex features in high dimensional datasets.

In order to further provide empirical evidence of the quality of our synthetic data, we rigorously interrogated the datasets using various statistical and mathematical methods to extract maximum information on any differential patterns between the two datasets. Prior to the tests, it was critical to test the normality of data features because many of the test methods utilized have underlying distributional assumptions—particularly normality. As such, the data were subjected to Kolmogorov–Smirnov and Shapiro–Wilk normality test and results revealed that all features in the datasets were drawn from a non-normally distributed population (Supplementary Table 3). Also, the datasets violated the homogeneity of variance assumption (i.e., have unequal variances) as well as had unequal sample sizes (Supplementary Tables 3 and 4). Consequent to this observation, our test methods were tempered accordingly as described subsequently.

The Welch’s and Brown-Forsythe tests were used to investigate if there is a significant difference between the means of two datasets. These tests are more reliable when the two samples have unequal variances and/or unequal sample sizes. The tests also assumes that the data groups are sampled from populations that follow a normal distribution. To this effect, the datasets were normalized using the Scikit-learn MinMax scaler algorithm. Both the Welch’s and Brown-Forsythe two-samples t-test showed that the difference in the means were statistically insignificant for all data features, time [statistic = 0.624, *p* = 0.437], temperature [statistic = 0.161, *p* = 0.692], culture amount [statistic = 0.086, *p* = 0.772], and alcohol content [statistic = 2.533, *p* = 0.125] (Supplementary Table 5). The Kruskal–Wallis H Test, which relies on the rank-ordering of data rather than on estimations involving variances and means was used to test the distributional similarity of the datasets by determining whether population medians were equal or not. This test is less sensitive to outliers and does not assume normality in the data. Results of the test indicated that the medians of both datasets are equal (*p* > 0.05) and that the samples originated from the same distribution (Supplementary Table 6).

Principal component analysis (PCA) and OPLS-DA were further employed to comparatively scrutinize the two datasets for inherent latent discriminatory patterns that might be elusive to conventional statistical analysis^59^. These models are capable of discriminating between high dimensional data groups via construction of condensed orthogonal variables called latent variables or principal components, essentially eigenvectors of the data's covariance matrix that explains the maximum variance from the variables. The first PCA analysis was used to examine the overall data structure, detect outliers, clusters, and data trends. The analysis showed two outliers, *i.e*., samples that were located outside of the Hotelling T2 ellipses (95% interval) on the score plot between PC1 and PC2. These data samples were excluded and PCs re-constructed. From the PCA analysis, the first two components of the PCA model explained 73.4% of the total variance in the datasets (PC1 = 43.6%, and PC2 = 29.8%). The PCA scores plot did not reveal any differential distribution patterns between the two datasets (Supplementary Fig. 1a). This plot (i.e., scores plot) provides a very visual representation of any inherent discriminatory patterns in datasets by showing sample clusters based on their dis/similarities in a projected two-dimensional space, defined by two of the selected principal components. Clearly, it can be seen that all the data points are randomly distributed around zero without any distinct clustering/groupings.

Since PCA does not supervise the construction of the latent variables (i.e., does not take into account the classification label associated with the data set), discrimination of the data groups may not be maximized. In this regard, OPLS-DA (a supervised multivariate data analysis technique) was used thus, yielding more class-specific discriminations of the datasets. According to Worley and Powers^63^, OPLS-DA aggressively separates experimental groups, as such it is frequently employed when PCA fails to reveal group separation. OPLS-DA has been employed to scrutinize and separate sample groups in complex and high dimensional datasets such as encountered in metabolomics and chemometrics^64,65^. OPLS-DA excels above PCA and other partial least squares discriminant analysis in group separation because it uses a single component to predict the class membership and the rest components to indicate variation orthogonal to the initial predictive component. Thus, OPLS-DA builds models that are more concise and interpretable, and is often employed to separate group-predictive and group-unrelated variance in the measured data^63^. In our study, the first two OPLS-DA models contained outliers, which were eliminated from the analysis, and a new OPLS-DA analysis was performed with all data points on/within the Hotelling T2 ellipses (95% interval). Results of the analysis aligned considerably with that of the PCA. The first two constructed latent variables accounted for 80% of the total explained variation *X* [i.e.*, R2*X(cum) = 0.80]. Despite the OPLS-DA model making reference to pre-defined sample class membership in order to maximize discrimination of the two data groups, there was no clear separation of the data groups (Supplementary Fig. 1b). No distinct sample clusters can be seen on the OPLS-DA scores plot. Variables from the two data groups can be seen randomly distributed across the graph. Overall, both the multivariate models (PCA and OPLS-DA) and the measures of distribution analysis (Welch’s test, Brown-Forsythe’s test, and Kruskal–Wallis H Test) provided good evidence that our GAN model synthesizes data that come from the true distribution.

### Predictive modelling of the fermentative production of bitter gourde-grape wine

A deep FCNN was constructed (Table 2) and trained using the synthetic data in order to model the relationship between the fermentation process conditions (temperature, time, and culture dosage) and the process outcome (alcohol content of bitter gourde-grape wine). The model consisted of five layers with a number of hyperparameters.

#### Hyperparameter tunning

Performance of ANNs depends necessarily on identifying a good set of hyperparameters—parameters whose values are used to regulate the model learning process^66^. It was thus necessary to tune the model hyperparameters in order to obtain the best suited model that most accurately describes our fermentation system. However, hyperparameter tunning is largely a non-trivial task as it entails optimizing non-convex and high-dimensional functions with unknown smoothness. Often, default hyperparameter values are chosen resulting in sub-optimal model performance^67^.

Herein, a hyperband algorithm^68^ was adopted for tunning hyperparameter values. This algorithm is specifically suited for large search spaces of discrete and continuous hyperparameters, especially when the computational cost to evaluate the performance of a set of hyperparameter configuration is high. The key advantage of hyperband is that it is adaptive in computation, allocating more resources (e.g., size of training set, number of features, or number of iterations for iterative algorithms) to promising hyperparameter configurations while promptly eliminating poorly performing configurations^68^. By adaptively allocating training resources, the algorithm is able to investigate orders-of-magnitude more hyperparameter configurations than conventional algorithms that uniformly train all configurations to completion, thus swiftly identifying good hyperparameters^67,68^. In fact, hyperband is 5–30 times faster than conventional Bayesian optimization methods. The following hyperparameters were tuned: number of layer units, activation functions, optimizer learning rate, and the dropout layer. The optimized hyperparameters for the model are presented in Table 3.

**Table 3:** Model hyperparameters.

S/No	Hyperparameter	Search space	Optimized condition
1	Number of units in input layer	Min (10), max (30), step (1)	11
2	Number of units in layer 4	Min (2), max (10), step (1)	6
3	Activation function for input layer	Values = softmax, relu, tanh, sigmoid, and linear	Softmax
4	Activation function for layer 4	Values = softmax, relu, tanh, sigmoid, and linear	Softmax
5	Activation function for output layer	Values = softmax, relu, tanh, sigmoid, and linear	Relu
6	Learning rate	Values = 0.01, 0.0001, and 1e-06	0.01
7	Dropout for layer 3	Min (0.5), max (0.7), step (0.1)	0.6
8	Dropout for layer 6	Min (0.2), max (0.5), step (0.1)	0.3

#### Model training

**Figure 5:**
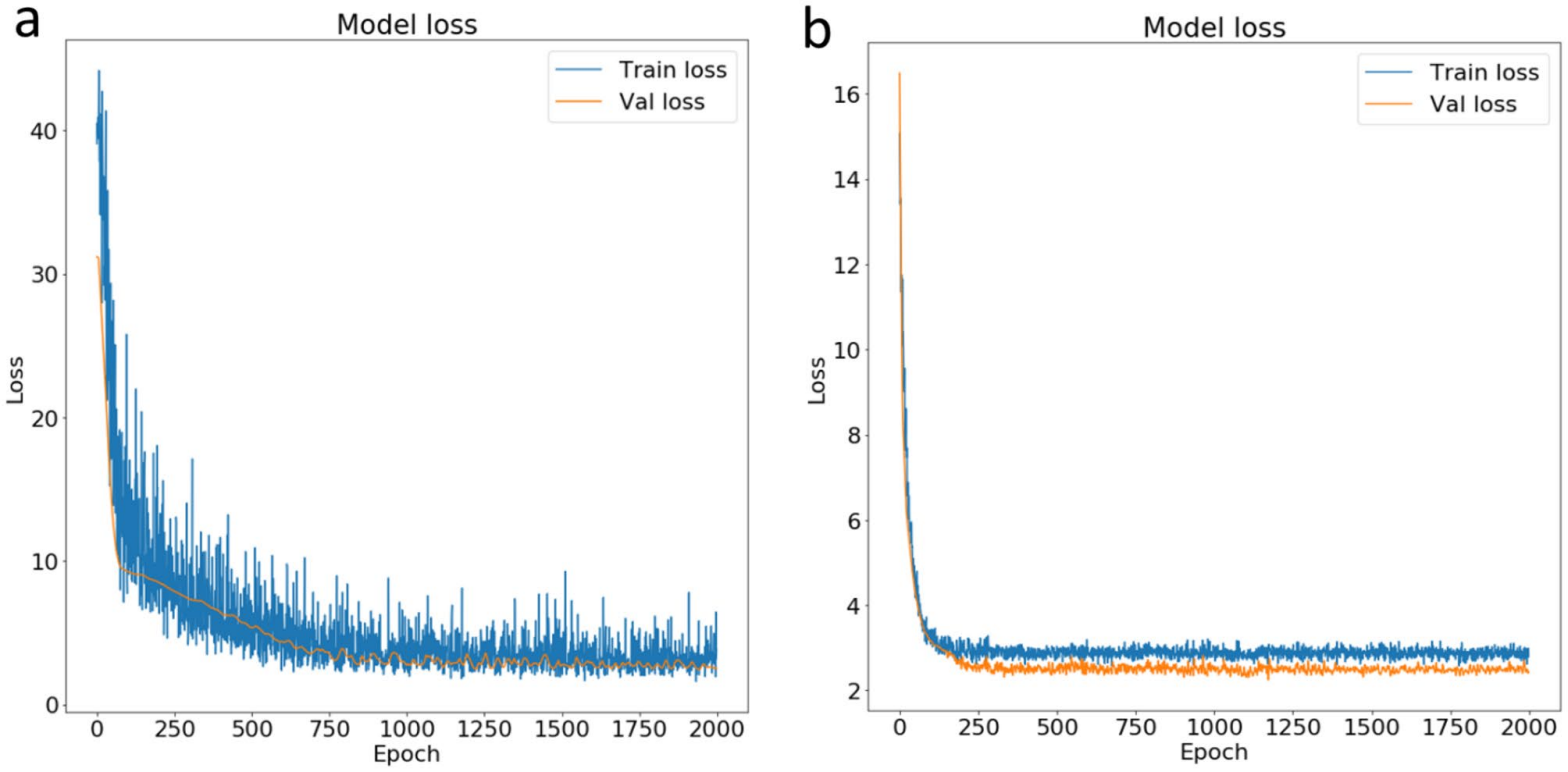
Model training and validation loss for (**a**) real data, and (**b**) synthetic data.

The optimized hyperparameters were used to initialize the model before training on the datasets. The training iteration was set at 2000 epochs with a batch size of 10. Both the real data and synthetic data were used to train the network. The performance of the model was evaluated using the model loss values. Since the model sought to minimize objective function via gradient descent, lower loss values indicate more accurate predictions. After training, the results show that the model trained with the synthetic data had a better learning progression and performance as compared to the model trained with the small experimental data (Fig. 5). The training sequence was repeated 10 different times and the average of the loss and metrics values recorded.

The model trained using the artificial data had a significantly (*p* ≤ 0.05) lower loss value (2.029 ± 0.124) and converged to a solution quicker as compared to its counterpart trained on the real data, which had a loss of 2.1614 ± 0.117. Interestingly, the opposite was the case for the results of the model evaluation metric. The performance metric (i.e., MSE) indicated that the model trained on the original data samples had a significantly (*p* ≤ 0.05) lower metric value of 0.804 ± 0.2042 than the model trained on the pseudo data (1.574 ± 0.069). Consequently, despite the tenfold inflation of the data, it was not possible to conclude that the model trained on the synthetic data was superior to its counterpart, nor was it possible to assert that the model trained on the real data is superior. One or the other may have fared better in an evaluation, depending on the measure used for the comparison (loss function or the metric). A possible reason for this could be because the synthetic data did not add any new information to the learning experience of the model.

Adequate training of an AI model encompasses not just quantity of data but also the quality of the data. The quality of a data could comprise quantity, diversity, accuracy, relevance, uniqueness, consistency, completeness, and validity amongst other characteristics. It is apparent that though more data was available to the model, the data fundamentally lacked diversity. According to Ansaldo^69^ too much data that does not have sufficient diversity and distribution to represent real-world problems can compromise model performance. The diversity of training data avails the model more discriminative information, allowing it to capture unique or complementary information during the learning phase. Additionally, if there is nothing new to learn from the data, even in situations where too much homogeneous/undiversified data has no effect on a trained model's predictive ability, it can result in costs in terms of time, power, and computer resources, among others, making the model more expensive and slower to build than it should be^69^. It was thus clear from the results of the model training that the quality of the data used to train a model is just as important as the quantity of the data.

#### Feature importance

Determination of the magnitude and significance of features is crucial to understand model behaviour and gain insight into the dynamics of the system being modeled, however, unlike simple linear models, this is not quite a straightforward task with “black box” models such as NNs. In this study, we adopted a permutation-based approach and shapely values to estimate the relative importance of each of our model features. The underlying principle of permutation feature importance or “Mean Decrease Accuracy (MDA)” is based on estimating the importance of a feature by calculating the decrease or increase in the model’s prediction error after permuting the feature. Thus, a feature is deemed unimportant if shuffling its values leaves the model error unchanged, because in this case, the model did not rely on the feature for the prediction. The opposite is the case for important features. The results of permutation feature importance showed that temperature was the most important feature (permutation weight = 0.7803 ± 0.2110) as it offered the most valuable information when predicting alcohol content in the model. Culture dosage (permutation weight = 0.0451 ± 0.0382) and then fermentation time (permutation weight = 0.0443 ± 0.0304) had almost similar permutation weights and were less important compared to fermentation temperature.

**Figure 6:**
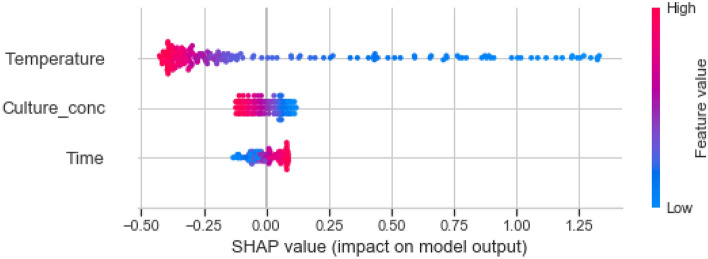
Combined shapely values for all model features.

In agreement with the results of permutation feature importance, the estimated shapely values showed that temperature had the highest relevance to model prediction of alcohol content, followed by starter culture dosage, and then fermentation time (Fig. 6). From the combined shapely values plot (i.e., Fig. 6), each row along the x-axis corresponds to a model feature and each dot represents a training sample. The colour gradient corresponds to the feature value, with red representing high feature value and blue representing low feature value. As can be observed from the plot, temperature is the feature with the highest impact on model predictive accuracy. This was already established when calculating the permutation importance. In addition to this insight, it can be deduced further that lower temperature values and longer fermentation times favours the production of alcohol in our fermentation system.

## Conclusion

Modelling of food processing operations (particularly fermentation processes) offers unique benefits such as providing increased understanding of system dynamics and predictive capabilities that can enhance food quality and improve food processing operations. Though very adaptive and able to learn complex relationships in process systems, AI models require quite a lot of training data in order to learn process patterns. This is a particular challenge for biological and biochemical systems such as fermentation because the process of obtaining such data is tedious, time consuming, and expensive. In this study, we describe the construction and generalization of a regression-type deep GAN architecture capable of synthesizing high-quality data from a relatively small sample of experimental data. We provide empirical evidence that our GAN model is able to explore the domain of real data configurations and tune model parameters to generate data that have similar properties as the experimental data. Moreover, the GAN model we defined herein can be conveniently generalized to any such (and possibly, all) regression problems. It was possible to train a DNN using the synthetic data. Training results were inconclusive as depending on the statistic used for comparison (loss function or the metric), the model trained on the artificial data, or its counterpart trained on the original data performed better than the other in evaluation. Limited diversity in the synthetic data was identified as the reason for the unimproved performance of the model trained on the pseudo data. Nonetheless, generalization of GANs to the regime of regression problems as herein described, offers peculiar advantages and opens several possibilities because currently, almost all proposed GAN models in the literature are designed for solving classification problems. Our study could therefore provide a framework for future researchers seeking to explore the previously unexploited domain of ANN modelling of biological systems limited majorly by data availability.

## Supplementary Information

Supplementary Table 1.

Supplementary Table 2.

Supplementary Table 3.

Supplementary Table 4.

Supplementary Table 5.

Supplementary Table 6.

Supplementary Figure 1.

## Data Availability

All data generated or analysed during this study are available within the article [and its supplementary material] and may also be made available upon reasonable request from the corresponding author, [S.G.].
