# Supplementary material for: Application of a generative adversarial network for multi-featured fermentation data synthesis and artificial neural network (ANN) modeling of bitter gourd–grape beverage production
Source: Sci Rep. 2023 Jul 20;13:11755. doi: 10.1038/s41598-023-38322-3 (PMC10359352; doi:10.1038/s41598-023-38322-3)
Supplement: Supplementary file 1 — Supplementary Table 1. [file 41598_2023_38322_MOESM1_ESM.docx]

Supplementary Table 1: Fermentation conditions and process parameters

| **S/No** | **Label** | **Data** | **Time (h)** | **Temperature (°C)** | **Culture dosage (v/v)** | **Alcohol (°P)** |
| --- | --- | --- | --- | --- | --- | --- |
|  | 1 | Real | 120.00 | 25.00 | 1.00 | 4.8 |
|  | 1 | Real | 72.00 | 45.11 | 3.00 | 11.8 |
|  | 1 | Real | 24.00 | 40.00 | 1.00 | 9.7 |
|  | 1 | Real | 120.00 | 40.00 | 1.00 | 7.7 |
|  | 1 | Real | 72.00 | 32.50 | 3.00 | 3.7 |
|  | 1 | Real | 24.00 | 40.00 | 5.00 | 9.5 |
|  | 1 | Real | 24.00 | 25.00 | 5.00 | 4.2 |
|  | 1 | Real | 120.00 | 25.00 | 5.00 | 4.6 |
|  | 1 | Real | 72.00 | 32.50 | 3.00 | 4.9 |
|  | 1 | Real | 152.73 | 32.50 | 3.00 | 4.9 |
|  | 1 | Real | 72.00 | 32.50 | 3.00 | 3.9 |
|  | 1 | Real | 72.00 | 19.89 | 3.00 | 4.8 |
|  | 1 | Real | 72.00 | 32.50 | 0.00 | 0.9 |
|  | 1 | Real | 72.00 | 32.50 | 6.36 | 3.9 |
|  | 1 | Real | 0.00 | 32.50 | 3.00 | 1 |
|  | 1 | Real | 72.00 | 32.50 | 3.00 | 3.6 |
|  | 1 | Real | 120.00 | 40.00 | 5.00 | 6.4 |
|  | 1 | Real | 72.00 | 32.50 | 3.00 | 3.7 |
|  | 1 | Real | 24.00 | 25.00 | 1.00 | 4.7 |
|  | 1 | Real | 72.00 | 32.50 | 3.00 | 3.7 |
