# Supplementary material for: Application of a generative adversarial network for multi-featured fermentation data synthesis and artificial neural network (ANN) modeling of bitter gourd–grape beverage production
Source: Sci Rep. 2023 Jul 20;13:11755. doi: 10.1038/s41598-023-38322-3 (PMC10359352; doi:10.1038/s41598-023-38322-3)
Supplement: Supplementary file 2 — Supplementary Table 2. [file 41598_2023_38322_MOESM2_ESM.docx]

Supplementary Table 2: Synthetic data produced by the GAN model

| **S/No** | **Label** | **Data** | **Time (h)** | **Temperature (°C)** | **Culture dosage (v/v)** | **Alcohol (°P)** |
| --- | --- | --- | --- | --- | --- | --- |
|  | 2 | Synthetic | 75.26 | 43.41 | 1.56 | 3.59 |
|  | 2 | Synthetic | 70.43 | 24.83 | 4.97 | 2.84 |
|  | 2 | Synthetic | 3.24 | 35.30 | 4.83 | 1.27 |
|  | 2 | Synthetic | 137.30 | 44.92 | 1.19 | 2.02 |
|  | 2 | Synthetic | 144.74 | 32.74 | 2.92 | 1.97 |
|  | 2 | Synthetic | 1.51 | 22.32 | 0.37 | 3.47 |
|  | 2 | Synthetic | 94.27 | 26.01 | 1.47 | 6.28 |
|  | 2 | Synthetic | 50.64 | 38.34 | 5.26 | 1.17 |
|  | 2 | Synthetic | 152.63 | 45.00 | 0.21 | 3.20 |
|  | 2 | Synthetic | 131.02 | 44.89 | 0.38 | 3.86 |
|  | 2 | Synthetic | 7.18 | 43.98 | 6.13 | 1.28 |
|  | 2 | Synthetic | 65.64 | 25.03 | 2.00 | 2.41 |
|  | 2 | Synthetic | 107.37 | 44.76 | 4.08 | 2.47 |
|  | 2 | Synthetic | 0.43 | 20.42 | 1.70 | 6.26 |
|  | 2 | Synthetic | 82.61 | 27.09 | 0.89 | 4.45 |
|  | 2 | Synthetic | 58.66 | 34.42 | 0.20 | 5.15 |
|  | 2 | Synthetic | 50.00 | 41.32 | 4.84 | 0.80 |
|  | 2 | Synthetic | 143.05 | 45.04 | 0.65 | 1.47 |
|  | 2 | Synthetic | 135.74 | 27.55 | 3.89 | 0.97 |
|  | 2 | Synthetic | 123.79 | 44.30 | 3.04 | 2.93 |
|  | 2 | Synthetic | 28.13 | 24.36 | 1.49 | 0.93 |
|  | 2 | Synthetic | 83.58 | 22.18 | 3.64 | 5.06 |
|  | 2 | Synthetic | 128.19 | 41.45 | 2.61 | 3.57 |
|  | 2 | Synthetic | 40.07 | 36.79 | 5.24 | 0.72 |
|  | 2 | Synthetic | 34.71 | 23.14 | 0.25 | 5.18 |
|  | 2 | Synthetic | 148.55 | 39.58 | 0.80 | 5.11 |
|  | 2 | Synthetic | 151.64 | 39.30 | 1.54 | 1.40 |
|  | 2 | Synthetic | 36.45 | 27.66 | 0.73 | 5.72 |
|  | 2 | Synthetic | 89.92 | 40.96 | 1.69 | 2.82 |
|  | 2 | Synthetic | 145.26 | 45.04 | 2.33 | 3.95 |
|  | 2 | Synthetic | 10.05 | 30.81 | 0.20 | 3.47 |
|  | 2 | Synthetic | 51.01 | 26.64 | 0.51 | 2.38 |
|  | 2 | Synthetic | 79.53 | 32.21 | 0.26 | 5.30 |
|  | 2 | Synthetic | 151.95 | 41.39 | 1.66 | 2.18 |
|  | 2 | Synthetic | 61.40 | 23.72 | 0.55 | 6.22 |
|  | 2 | Synthetic | 10.50 | 23.54 | 0.44 | 5.02 |
|  | 2 | Synthetic | 29.59 | 27.97 | 5.49 | 0.44 |
|  | 2 | Synthetic | 129.83 | 40.56 | 1.16 | 3.03 |
|  | 2 | Synthetic | 71.90 | 25.57 | 2.19 | 1.56 |
|  | 2 | Synthetic | 2.03 | 20.08 | 5.12 | 4.92 |
|  | 2 | Synthetic | 62.76 | 32.93 | 5.77 | 0.94 |
|  | 2 | Synthetic | 93.31 | 27.48 | 4.95 | 3.35 |
|  | 2 | Synthetic | 56.00 | 21.02 | 2.45 | 5.56 |
|  | 2 | Synthetic | 32.46 | 40.39 | 0.42 | 4.33 |
|  | 2 | Synthetic | 121.87 | 32.62 | 3.08 | 2.61 |
|  | 2 | Synthetic | 47.47 | 38.50 | 2.21 | 1.01 |
|  | 2 | Synthetic | 32.57 | 40.97 | 5.69 | 0.69 |
|  | 2 | Synthetic | 67.84 | 36.48 | 0.73 | 4.24 |
|  | 2 | Synthetic | 99.96 | 28.40 | 0.87 | 4.27 |
|  | 2 | Synthetic | 148.77 | 42.01 | 3.50 | 2.59 |
|  | 2 | Synthetic | 152.31 | 44.41 | 0.49 | 1.43 |
|  | 2 | Synthetic | 144.80 | 44.74 | 0.56 | 1.52 |
|  | 2 | Synthetic | 7.26 | 20.64 | 0.73 | 5.85 |
|  | 2 | Synthetic | 108.29 | 42.41 | 2.74 | 0.83 |
|  | 2 | Synthetic | 57.87 | 38.20 | 2.87 | 0.62 |
|  | 2 | Synthetic | 57.19 | 42.50 | 4.81 | 0.91 |
|  | 2 | Synthetic | 149.24 | 40.42 | 1.52 | 1.91 |
|  | 2 | Synthetic | 84.46 | 23.41 | 1.95 | 3.38 |
|  | 2 | Synthetic | 133.74 | 43.95 | 0.38 | 2.78 |
|  | 2 | Synthetic | 28.99 | 24.58 | 5.81 | 4.81 |
|  | 2 | Synthetic | 7.42 | 22.43 | 5.78 | 0.32 |
|  | 2 | Synthetic | 149.99 | 41.20 | 0.83 | 1.98 |
|  | 2 | Synthetic | 121.95 | 28.92 | 1.41 | 5.17 |
|  | 2 | Synthetic | 146.57 | 31.15 | 3.12 | 3.61 |
|  | 2 | Synthetic | 149.47 | 28.92 | 4.75 | 4.60 |
|  | 2 | Synthetic | 15.20 | 43.08 | 5.68 | 2.48 |
|  | 2 | Synthetic | 145.07 | 32.19 | 4.77 | 4.13 |
|  | 2 | Synthetic | 1.90 | 20.45 | 5.27 | 3.66 |
|  | 2 | Synthetic | 74.04 | 24.05 | 4.63 | 3.70 |
|  | 2 | Synthetic | 78.87 | 24.49 | 1.14 | 4.53 |
|  | 2 | Synthetic | 115.96 | 25.27 | 4.30 | 2.44 |
|  | 2 | Synthetic | 14.02 | 34.87 | 6.21 | 0.61 |
|  | 2 | Synthetic | 150.01 | 39.26 | 2.69 | 3.09 |
|  | 2 | Synthetic | 93.89 | 42.34 | 0.43 | 2.11 |
|  | 2 | Synthetic | 137.69 | 40.58 | 4.73 | 3.87 |
|  | 2 | Synthetic | 139.91 | 31.43 | 3.45 | 4.16 |
|  | 2 | Synthetic | 111.54 | 44.16 | 5.96 | 3.12 |
|  | 2 | Synthetic | 55.76 | 29.81 | 3.82 | 4.76 |
|  | 2 | Synthetic | 122.79 | 27.95 | 3.68 | 2.70 |
|  | 2 | Synthetic | 113.49 | 44.76 | 1.47 | 1.67 |
|  | 2 | Synthetic | 129.52 | 39.87 | 4.72 | 2.04 |
|  | 2 | Synthetic | 65.25 | 41.37 | 2.77 | 1.95 |
|  | 2 | Synthetic | 124.21 | 34.39 | 3.30 | 3.79 |
|  | 2 | Synthetic | 143.02 | 38.83 | 1.55 | 2.78 |
|  | 2 | Synthetic | 59.73 | 25.75 | 4.53 | 2.61 |
|  | 2 | Synthetic | 152.34 | 44.79 | 0.34 | 2.21 |
|  | 2 | Synthetic | 131.78 | 30.21 | 5.54 | 4.41 |
|  | 2 | Synthetic | 43.36 | 24.60 | 2.36 | 0.82 |
|  | 2 | Synthetic | 19.27 | 20.48 | 3.79 | 6.29 |
|  | 2 | Synthetic | 123.27 | 43.96 | 1.50 | 2.19 |
|  | 2 | Synthetic | 73.43 | 23.33 | 1.43 | 5.69 |
|  | 2 | Synthetic | 123.28 | 26.13 | 1.11 | 1.69 |
|  | 2 | Synthetic | 56.30 | 36.29 | 5.46 | 2.04 |
|  | 2 | Synthetic | 75.10 | 31.78 | 1.24 | 5.22 |
|  | 2 | Synthetic | 46.53 | 32.63 | 4.17 | 0.25 |
|  | 2 | Synthetic | 11.30 | 21.85 | 2.28 | 3.41 |
|  | 2 | Synthetic | 19.82 | 24.12 | 2.26 | 4.43 |
|  | 2 | Synthetic | 9.44 | 21.46 | 1.17 | 6.17 |
|  | 2 | Synthetic | 122.44 | 39.79 | 4.76 | 1.47 |
|  | 2 | Synthetic | 49.60 | 34.01 | 5.61 | 2.20 |
|  | 2 | Synthetic | 150.90 | 41.74 | 2.65 | 1.39 |
|  | 2 | Synthetic | 39.37 | 22.65 | 6.18 | 5.70 |
|  | 2 | Synthetic | 16.54 | 29.57 | 5.64 | 0.50 |
|  | 2 | Synthetic | 124.09 | 39.49 | 4.13 | 1.25 |
|  | 2 | Synthetic | 77.74 | 22.72 | 6.20 | 3.69 |
|  | 2 | Synthetic | 83.77 | 26.62 | 2.46 | 4.18 |
|  | 2 | Synthetic | 140.02 | 33.27 | 1.74 | 5.70 |
|  | 2 | Synthetic | 120.07 | 27.53 | 2.81 | 3.42 |
|  | 2 | Synthetic | 38.72 | 21.58 | 2.97 | 5.93 |
|  | 2 | Synthetic | 3.31 | 20.23 | 6.17 | 5.18 |
|  | 2 | Synthetic | 140.90 | 41.56 | 4.15 | 4.30 |
|  | 2 | Synthetic | 110.06 | 26.95 | 3.76 | 4.84 |
|  | 2 | Synthetic | 13.63 | 30.28 | 1.47 | 1.36 |
|  | 2 | Synthetic | 129.86 | 44.83 | 1.67 | 2.32 |
|  | 2 | Synthetic | 4.39 | 25.58 | 1.10 | 1.93 |
|  | 2 | Synthetic | 18.22 | 23.86 | 5.32 | 5.45 |
|  | 2 | Synthetic | 104.01 | 29.92 | 4.26 | 0.81 |
|  | 2 | Synthetic | 41.56 | 36.81 | 5.94 | 2.74 |
|  | 2 | Synthetic | 145.97 | 41.79 | 1.62 | 2.39 |
|  | 2 | Synthetic | 39.43 | 38.73 | 1.66 | 2.52 |
|  | 2 | Synthetic | 0.62 | 20.99 | 0.33 | 5.62 |
|  | 2 | Synthetic | 48.07 | 36.70 | 5.65 | 6.22 |
|  | 2 | Synthetic | 141.92 | 44.64 | 3.31 | 2.30 |
|  | 2 | Synthetic | 55.37 | 25.84 | 5.54 | 1.60 |
|  | 2 | Synthetic | 94.20 | 44.14 | 6.18 | 3.65 |
|  | 2 | Synthetic | 147.61 | 40.72 | 2.20 | 1.61 |
|  | 2 | Synthetic | 115.08 | 27.21 | 5.47 | 5.67 |
|  | 2 | Synthetic | 114.62 | 30.95 | 4.43 | 1.71 |
|  | 2 | Synthetic | 119.92 | 36.03 | 3.21 | 2.69 |
|  | 2 | Synthetic | 47.84 | 41.34 | 2.35 | 2.99 |
|  | 2 | Synthetic | 3.49 | 22.32 | 3.70 | 4.79 |
|  | 2 | Synthetic | 0.61 | 22.41 | 0.15 | 5.95 |
|  | 2 | Synthetic | 92.91 | 44.81 | 2.27 | 1.50 |
|  | 2 | Synthetic | 23.97 | 40.04 | 4.96 | 1.64 |
|  | 2 | Synthetic | 62.07 | 24.47 | 1.70 | 3.70 |
|  | 2 | Synthetic | 2.75 | 20.64 | 4.10 | 6.04 |
|  | 2 | Synthetic | 6.18 | 26.47 | 0.33 | 5.12 |
|  | 2 | Synthetic | 75.06 | 28.42 | 0.68 | 2.34 |
|  | 2 | Synthetic | 143.02 | 42.76 | 5.44 | 3.52 |
|  | 2 | Synthetic | 147.97 | 39.85 | 2.01 | 3.42 |
|  | 2 | Synthetic | 127.87 | 44.98 | 1.64 | 2.89 |
|  | 2 | Synthetic | 17.06 | 39.94 | 5.13 | 1.97 |
|  | 2 | Synthetic | 72.90 | 26.13 | 2.06 | 6.12 |
|  | 2 | Synthetic | 103.79 | 32.31 | 2.00 | 1.42 |
|  | 2 | Synthetic | 11.97 | 20.78 | 6.22 | 1.87 |
|  | 2 | Synthetic | 45.22 | 26.94 | 4.07 | 1.76 |
|  | 2 | Synthetic | 119.97 | 43.86 | 1.28 | 3.32 |
|  | 2 | Synthetic | 93.66 | 33.86 | 0.48 | 4.90 |
|  | 2 | Synthetic | 124.20 | 36.03 | 1.02 | 3.75 |
|  | 2 | Synthetic | 148.95 | 41.43 | 1.63 | 2.16 |
|  | 2 | Synthetic | 15.48 | 21.67 | 6.22 | 5.83 |
|  | 2 | Synthetic | 66.09 | 23.65 | 5.21 | 2.40 |
|  | 2 | Synthetic | 48.22 | 40.15 | 5.16 | 3.25 |
|  | 2 | Synthetic | 32.88 | 29.33 | 4.87 | 0.51 |
|  | 2 | Synthetic | 83.96 | 41.35 | 3.42 | 1.39 |
|  | 2 | Synthetic | 129.05 | 27.95 | 3.55 | 1.43 |
|  | 2 | Synthetic | 152.17 | 42.29 | 1.21 | 2.62 |
|  | 2 | Synthetic | 63.75 | 33.97 | 3.89 | 2.57 |
|  | 2 | Synthetic | 128.82 | 44.73 | 4.88 | 2.21 |
|  | 2 | Synthetic | 144.59 | 45.02 | 1.26 | 3.29 |
|  | 2 | Synthetic | 72.83 | 28.44 | 3.95 | 4.44 |
|  | 2 | Synthetic | 14.25 | 39.91 | 5.42 | 0.45 |
|  | 2 | Synthetic | 3.13 | 20.10 | 6.06 | 6.21 |
|  | 2 | Synthetic | 144.25 | 36.89 | 5.17 | 2.34 |
|  | 2 | Synthetic | 152.19 | 43.62 | 1.37 | 2.66 |
|  | 2 | Synthetic | 145.62 | 39.00 | 4.75 | 5.96 |
|  | 2 | Synthetic | 47.44 | 22.78 | 5.45 | 6.30 |
|  | 2 | Synthetic | 44.56 | 33.88 | 0.35 | 5.01 |
|  | 2 | Synthetic | 70.42 | 30.70 | 5.38 | 0.45 |
|  | 2 | Synthetic | 117.06 | 24.35 | 4.03 | 4.34 |
|  | 2 | Synthetic | 136.77 | 38.04 | 5.53 | 5.94 |
|  | 2 | Synthetic | 31.61 | 34.07 | 6.27 | 2.69 |
|  | 2 | Synthetic | 118.70 | 25.97 | 1.99 | 6.32 |
|  | 2 | Synthetic | 45.80 | 44.83 | 3.49 | 2.59 |
|  | 2 | Synthetic | 143.09 | 36.57 | 2.95 | 2.77 |
|  | 2 | Synthetic | 57.04 | 29.90 | 6.19 | 1.53 |
|  | 2 | Synthetic | 11.33 | 23.23 | 4.38 | 0.81 |
|  | 2 | Synthetic | 148.98 | 34.94 | 1.36 | 4.61 |
|  | 2 | Synthetic | 21.90 | 28.02 | 1.57 | 3.95 |
|  | 2 | Synthetic | 4.62 | 22.86 | 4.25 | 5.60 |
|  | 2 | Synthetic | 89.33 | 32.51 | 1.48 | 3.26 |
|  | 2 | Synthetic | 74.57 | 36.45 | 1.11 | 3.77 |
|  | 2 | Synthetic | 20.29 | 23.11 | 5.81 | 3.89 |
|  | 2 | Synthetic | 135.23 | 29.74 | 2.24 | 1.87 |
|  | 2 | Synthetic | 152.32 | 43.45 | 1.26 | 2.40 |
|  | 2 | Synthetic | 3.22 | 20.10 | 6.19 | 5.83 |
|  | 2 | Synthetic | 43.71 | 26.84 | 2.41 | 4.67 |
|  | 2 | Synthetic | 77.74 | 24.57 | 5.26 | 5.49 |
|  | 2 | Synthetic | 127.04 | 37.29 | 2.91 | 3.29 |
|  | 2 | Synthetic | 151.95 | 44.47 | 1.11 | 3.81 |
|  | 2 | Synthetic | 23.37 | 43.82 | 5.78 | 1.42 |
|  | 2 | Synthetic | 3.72 | 21.30 | 5.96 | 2.16 |
|  | 2 | Synthetic | 71.94 | 28.48 | 6.08 | 3.71 |
|  | 2 | Synthetic | 37.54 | 22.13 | 3.74 | 2.29 |
|  | 2 | Synthetic | 72.93 | 27.89 | 5.15 | 3.03 |
|  | 2 | Synthetic | 41.75 | 44.26 | 6.23 | 3.25 |
|  | 2 | Synthetic | 124.83 | 37.66 | 2.62 | 0.98 |
|  | 2 | Synthetic | 88.45 | 44.99 | 2.71 | 3.18 |
|  | 2 | Synthetic | 22.26 | 41.90 | 5.99 | 0.86 |
|  | 2 | Synthetic | 91.64 | 38.56 | 4.50 | 2.10 |
