# Supplementary material for: Application of a generative adversarial network for multi-featured fermentation data synthesis and artificial neural network (ANN) modeling of bitter gourd–grape beverage production
Source: Sci Rep. 2023 Jul 20;13:11755. doi: 10.1038/s41598-023-38322-3 (PMC10359352; doi:10.1038/s41598-023-38322-3)
Supplement: Supplementary file 3 — Supplementary Table 3. [file 41598_2023_38322_MOESM3_ESM.docx]

Supplementary Table 3: Kolmogorov-Smirnov and Shapiro-Wilk’s test of normality

| **Variable** | **Real data** | | | | | | **Synthetic data** | | | | | |
| --- | --- | --- | --- | --- | --- | --- | --- | --- | --- | --- | --- | --- |
|  | **Kolmogorov-Smirnov^a^** | | | **Shapiro-Wilk** | | | **Kolmogorov-Smirnov^a^** | | | **Shapiro-Wilk** | | |
|  | **Statistic** | **df** | **Sig.** | **Statistic** | **df** | **Sig.** | **Statistic** | **df** | **Sig.** | **Statistic** | **df** | **Sig.** |
| Time | .254 | 20 | .001 | .892 | 20 | .029 | .120 | 200 | .000 | .921 | .921 | .000 |
| Temperature | .250 | 20 | .002 | .897 | 20 | .037 | .111 | 200 | .000 | .918 | .918 | .000 |
| Culture dosage | .254 | 20 | .001 | .892 | 20 | .029 | .116 | 200 | .000 | .928 | .928 | .000 |
| Alcohol | .282 | 20 | .000 | .876 | 20 | .015 | .072 | 200 | .013 | .960 | .960 | .000 |

Key: a=Lilliefors Significance Correction
