# Supplementary material for: Application of a generative adversarial network for multi-featured fermentation data synthesis and artificial neural network (ANN) modeling of bitter gourd–grape beverage production
Source: Sci Rep. 2023 Jul 20;13:11755. doi: 10.1038/s41598-023-38322-3 (PMC10359352; doi:10.1038/s41598-023-38322-3)
Supplement: Supplementary file 4 — Supplementary Table 4. [file 41598_2023_38322_MOESM4_ESM.docx]

Supplementary Table 4: Test of Homogeneity of Variances

| **Variable** | **Levene Statistic** | **df1** | **df2** | **Sig.** |
| --- | --- | --- | --- | --- |
| Time | 9.558 | 1 | 218 | .002 |
| Temperature | 12.942 | 1 | 218 | .000 |
| Culture dosage | 7.913 | 1 | 218 | .005 |
| Alcohol | 5.180 | 1 | 218 | .024 |
