# Supplementary material for: Application of a generative adversarial network for multi-featured fermentation data synthesis and artificial neural network (ANN) modeling of bitter gourd–grape beverage production
Source: Sci Rep. 2023 Jul 20;13:11755. doi: 10.1038/s41598-023-38322-3 (PMC10359352; doi:10.1038/s41598-023-38322-3)
Supplement: Supplementary file 5 — Supplementary Table 5. [file 41598_2023_38322_MOESM5_ESM.docx]

Supplementary Table 5: Robust Tests of Equality of Means

| **Variable** | | **Statistic^a^** | **df1** | **df2** | **Sig.** |
| --- | --- | --- | --- | --- | --- |
| Time | Welch | 0.624 | 1 | 25.542 | 0.437 |
|  | Brown-Forsythe | 0.624 | 1 | 25.542 | 0.437 |
| Temperature | Welch | 0.161 | 1 | 25.944 | 0.692 |
|  | Brown-Forsythe | 0.161 | 1 | 25.944 | 0.692 |
| Culture dosage | Welch | 0.086 | 1 | 24.925 | 0.772 |
|  | Brown-Forsythe | 0.086 | 1 | 24.925 | 0.772 |
| Alcohol | Welch | 2.533 | 1 | 23.876 | 0.125 |
|  | Brown-Forsythe | 2.533 | 1 | 23.876 | 0.125 |

Key: a=Asymptotically F distributed.
