# Supplementary material for: Application of a generative adversarial network for multi-featured fermentation data synthesis and artificial neural network (ANN) modeling of bitter gourd–grape beverage production
Source: Sci Rep. 2023 Jul 20;13:11755. doi: 10.1038/s41598-023-38322-3 (PMC10359352; doi:10.1038/s41598-023-38322-3)
Supplement: Supplementary file 6 — Supplementary Table 6. [file 41598_2023_38322_MOESM6_ESM.docx]

Supplementary Table 6: Kruskal-Wallis H Test of real and synthetic data

| **Ranks** | | | | **Test Statistics^a,b^** | | |
| --- | --- | --- | --- | --- | --- | --- |
| **Variable** | **Group** | **N** | **Mean Rank** | **Chi-Square** | **df** | **Asymp. Sig.** |
| Time | Fake | 200 | 111.40 | 0.440 | 1 | 0.507 |
|  | Real | 20 | 101.50 |  |  |  |
|  | Total | 220 |  |  |  |  |
| Temperature | Fake | 200 | 110.89 | 0.083 | 1 | 0.774 |
|  | Real | 20 | 106.60 |  |  |  |
|  | Total | 220 |  |  |  |  |
| Culture dosage | Fake | 200 | 110.70 | 0.022 | 1 | 0.883 |
|  | Real | 20 | 108.50 |  |  |  |
|  | Total | 220 |  |  |  |  |
| Alcohol | Fake | 200 | 112.54 | 2.260 | 1 | 0.133 |
|  | Real | 20 | 90.10 |  |  |  |
|  | Total | 220 |  |  |  |  |

Key: a=Kruskal Wallis test; b=grouping variable: group
