# Supplementary material for: Application of a generative adversarial network for multi-featured fermentation data synthesis and artificial neural network (ANN) modeling of bitter gourd–grape beverage production
Source: Sci Rep. 2023 Jul 20;13:11755. doi: 10.1038/s41598-023-38322-3 (PMC10359352; doi:10.1038/s41598-023-38322-3)

Supplementary Figure 1: Supervised and unsupervised linear transformation and multivariate dimensional reduction analysis of the real and synthetic data: (a) PCA and (b) OPLS-DA.


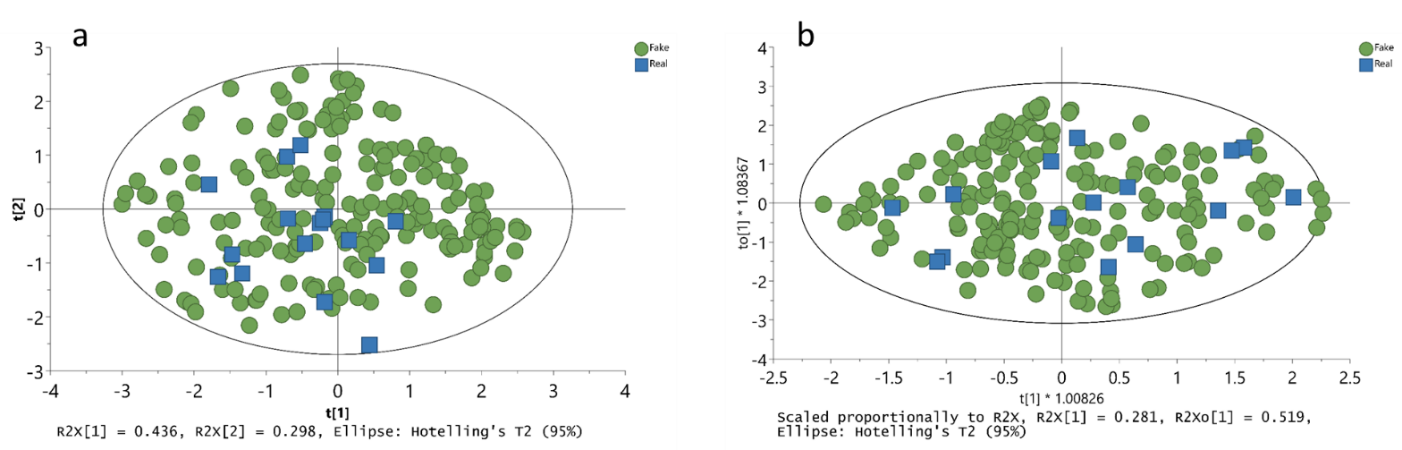

Supplement: Supplementary file 7 — Supplementary Figure 1. [file 41598_2023_38322_MOESM7_ESM.docx]
